# Non-porous silica nanoparticles as a cavitation sensitive vehicle for antibiotic delivery

**DOI:** 10.1016/j.ultsonch.2025.107316

**Published:** 2025-03-17

**Authors:** Grace Ball, Jack Stevenson, Faraz Amini Boroujeni, Ben Jacobson, Sarah A. Kuehne, Margaret Lucas, Anthony Damien Walmsley, Paul Prentice, Zoe Pikramenou

**Affiliations:** a School of Chemistry, University of Birmingham, Edgbaston B15 2TT, UK; b Centre for Medical & Industrial Ultrasonics, James Watt School of Engineering, University of Glasgow, Glasgow G12 8QQ, UK; c School of Science & Technology, Nottingham Trent University, Nottingham NG11 8NS, UK; d School of Dentistry, College of Medical and Dental Sciences, University of Birmingham, Birmingham B5 7EG, UK

**Keywords:** Ultrasound, Cavitation responsive, Silica nanoparticles, Controlled drug release

## Abstract

Ultrasound stimulated drug delivery is attractive for controlled dose and localised delivery to reduce excess loss of drug and side effects, which for antibiotics is pertinent to the fight against antimicrobial resistance. Low frequency ultrasound is commonly used in dental clinical practice for bacterial biofilm removal and is an attractive versatile stimulus for drug release. Here we introduce nonporous (amorphous) silica nanoparticles as a biocompatible, encapsulant for triggered drug release by low frequency ultrasound. A 20 kHz ultrasonic sonotrode is used in to evaluate the release of the antibiotic ciprofloxacin, CPX, from non-porous particles, **CPX ⊂ SiO_2_**. Laser doppler vibrometry (LDV) was employed to characterise the ultrasonic vibration displacement of the sonotrode. Drug release from **CPX ⊂ SiO_2_** was monitored for increasing the tip displacement. Clinically relevant quantities of CPX release (5.7 mg/L) occurred at 40 μm tip displacement in our studies. A strong correlation was observed between cavitation features in the acoustic spectra and drug release from **CPX ⊂ SiO_2_**. Silica nanoparticles with and without encapsulated CPX, **CPX ⊂ SiO_2_** and **SiO_2_**, respectively, were found to promote cavitation at lower amplitudes confirmed by high-speed imaging, in contrast to mesoporous particles with and without adsorbed CPX, **CPX@m-SiO_2_** and **m-SiO_2_**. Spectra of the emissions collected via an acoustic cavitation detector supported these results. Our studies demonstrate a novel platform for drug delivery employing low frequency ultrasound for synergistic enhancement of cavitation effects and triggered drug release.

## Highlights

• LDV measurements characterise a 20 kHz sonotrode.
• 20 kHz ultrasound enables controlled antibiotic release from non-porous silica nanoparticles.
• Non-porous silica nanoparticles enhance cavitation, deduced by high-speed imaging and acoustic detection.

## Graphical abstract

**Figure:**
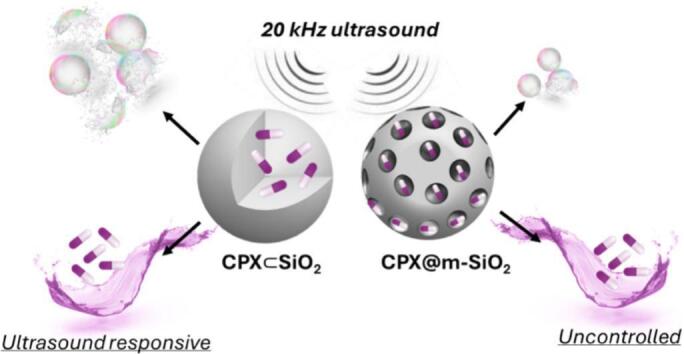

## 1 Introduction

Ultrasound is widely used in the clinic for a variety of applications including imaging, diagnostics, and more recently in therapeutic applications. Drug delivery using ultrasound has attracted attention using both high frequency (>1 MHz) and low frequency (20–100 kHz) ultrasound [1], [2], [3]. Microbubbles, pluronic micelles, inorganic nanoparticles among others, have all been explored as drug carriers with site selective delivery by ultrasound mediated effects [4], [5], [6], [7]. Drug delivery approaches using ultrasound may include microbubbles with high payloads although nanosized carriers have the advantage of smaller size for hard to reach areas and open new possibilities for precise, localised targeted delivery [3]. A challenge in the drug delivery systems is the trigger for the drug to be released and in the majority of the studies this is based on a chemical reaction hence high frequency, focused ultrasound has been used. The production of reactive oxygen species is also a major role in high frequency ultrasound for therapeutic treatments. Drugs may also be detached by the surface of nanosized carriers due to cavitation processes [8]. Cavitation is enabled by ultrasound based on the periodic growth of gas-filled bubbles in solution. Cavitating bubbles can be described as non-inertial and inertial, where non-inertial bubbles oscillate without collapse, generating relatively mild effects such as microstreaming, with inertial cavitation involving the rapid collapse of bubbles, leading to high energy shock waves and jetting. The effects of the presence of nanoparticles on the cavitation response of MHz ultrasonic transducers have been studied extensively. [9] Cavitation produces high pressures that may lead to risk of damage to surrounding tissues and this may restrict how much energy is applied over time [10]. Microbubbles [11] and more recently silica nanoparticles [12], [13] have shown to facilitate cavitation at lower pressures. Even though cavitation is more pronounced in low frequencies the physical effects of nanoparticles for drug release in the presence of low frequency ultrasound have not been thoroughly studied [14], [15], [16], [17] possibly due to the lack of necessity to reduce onset pressure. Low frequency ultrasound (20–100 kHz) has found extensive use in clinical treatments mainly in enhanced transdermal drug delivery, due to the cavitating bubbles enhancing tissue penetration of drug molecules, namely sonophoresis [13], [14]. It is also used in dental biofilm removal, wound debridement [18] thrombolytic technology [19] and in surgery including bone cutting and tumour ablation [15], [16], [17].

Silica nanoparticles are popular drug carriers based on their biocompatibility, ease of synthesis, and chemical modification [15], [16], [20]. Mesoporous silica nanoparticles (**m-SiO_2_**) have attracted the most attention, where their porous structure, with defined porous channels (width 2–50 nm), enables the adsorption of drugs. However, uncontrolled drug release from **m-SiO_2_** upon their suspension in fluid media prevents site selective delivery and can only be bypassed by further chemical functionalisation of the outside of the silica surface to cap the pores or formation of outer shell [[21], [22]]. We have previously introduced amorphous, silica nanoparticles (**SiO_2_**) which do not have an organised porous network to encapsulate drug molecules into the nanoparticle framework during the framework formation [[23], [24], [25]]. This approach prevents uncontrolled release and increases the potential antibiotic choice for localised delivery. Amorphous, non-porous silica nanoparticles provide a rough surface which can lead to nucleation for bubble formation and cavitation being a promising carrier material with for ultrasound selective release [[26], [27], [28]]. The mechanism of drug release from amorphous, non-porous silica nanoparticles using kHz ultrasound has not been previously explored.

Ciprofloxacin (CPX) was chosen for encapsulation into non-porous silica nanoparticles due to its applications in treatment of skin and bone infections, where previously CPX has been shown to have enhanced antibacterial activity with applied low frequency ultrasound due to sonophoresis [[29], [30], [31], [32]]. CPX is a broad spectrum antibiotic; it is active against both Gram-negative and Gram-positive bacteria, killing bacteria by inhibiting DNA gyrase, preventing DNA replication [33]. Combining the effects of low frequency sonophoresis and site selective drug delivery from ultrasound triggered silica nanoparticles would contribute to tackling the issue of emerging bacterial resistance and potentially enhance the treatment of chronic wound infections [34]. Localised drug delivery can allow for more effective bacterial eradication as well as preventing off-target effects.

**Fig. 1:**
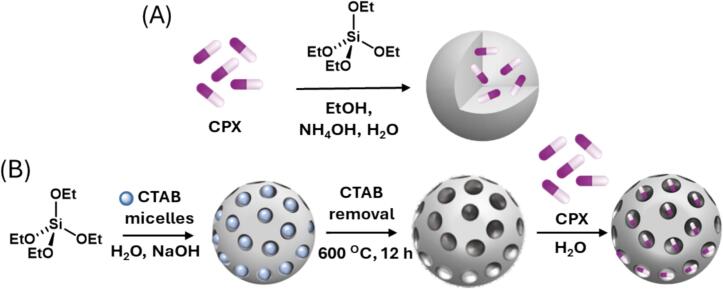
Schematic representation of (A) non-porous silica nanoparticles with CPX encapsulated (CPX ⊂ SiO_2_) and (B) mesoporous silica nanoparticles with CPX absorbed (CPX-m-SiO_2_).

In this study we use Laser doppler vibrometry (LDV) to measure the tip displacement amplitude generated by a 20 kHz sonotrode. Non-porous silica nanoparticles with CPX encapsulated (**CPX ⊂ SiO_2_**) and mesoporous silica nanoparticles with CPX absorbed (**CPX-m-SiO_2_**) are synthesised for comparison of drug release properties in the presence of ultrasound (Fig. 1). We use a shockwave passive cavitation detector (swPCD) [35] to analyse the cavitation effects of non-porous silica nanoparticles and mesoporous silica nanoparticles with and without CPX.

## 2 Experimental

### 2.1 Materials and instrumentation

Ciprofloxacin (CPX) was purchased from Fluorochem. Deionised water is used in all measurements. The remainder of the chemicals and consumables were purchased from Sigma-Aldrich. Dynamic Light Scattering (DLS) data were recorded using a Malvern Panalytical Zetasizer ZS instrument equipped with a He-Ne 633 nm laser at 25 °C using MilliQ water as dispersant. Data were collected with Malvern DTS 7.03 software. All sizes were determined based on 5 measurements each with 10 accumulations with a run length of 10 s each. Volume-averaged values, determined by the Zetasizer software based on Mie theory, were used. Mean values give the average of 5 different measurements. ζ-potential was determined by three measurements at 25 °C for each sample with >50 runs each, combining electrophoretic mobility and laser doppler vibrometry with an applied potential of ±150 V. Solid-state UV–Vis spectra were recorded using a Cary-5000 UV–Vis spectrophotometer equipped with an integrating sphere. Steady state luminescence measurements and time resolved studies were recorded on an Edinburgh Instruments FLS1000 steady state and time-resolved spectrometer equipped with Fluoracle software. All spectra were corrected for photomultiplier (Hamamatsu R928) and instrumental response. Luminescent lifetimes were recorded using an EPL-375 laser as an excitation source and fitted using Edinburgh Instruments FAST software with a calculated error of ±0.1 ns. Analysis of supernatant after synthesis of particles and after application of ultrasound was carried out using a Cary60 UV–Vis spectrometer. Ultrasound drug release measurements were carried out in triplicate. Transmission electron microscopy (TEM) was performed using a JEOL-2100 microscope with the sample loaded onto copper carbon formvar grids with a 200-mesh size. Images were processed in ImageJ software. The sonotrode was a Fisherbrand Model 120 operating at 20 kHz with a 3 mm probe diameter, input power was given as a reading (W) on the control console when the probe was immersed in solution.

### 2.2 Synthesis of SiO_2_

Silica nanoparticles (**SiO_2_**) were synthesised for control experiments without antibiotic using adapted amorphous **SiO_2_** synthesis reported by L. M. Rossi *et al* [36]. A solution of EtOH (25 mL), NH_4_OH (1.52 mL of 28 % w/v, 0.9 M) and H_2_O (125 μL, 0.28 M) was stirred and tetraethyl orthosilicate (TEOS) (2 mL in 5 mL EtOH, d = 0.933 gml^−1^, 9 mmol) was added dropwise to the solution at room temperature. The resulting solution was stirred for 2 h at room temperature, followed by 15 min sonication (200 W, 20 kHz), then another 1 h of stirring. The white suspension was then centrifuged (7450 rpm, 15 min) and the supernatant was removed. Resulting nanoparticles were then washed with H_2_O (3 x 25 mL), dried under vacuum and isolated as a white powder (0.64 g); diameter 124 ± 25 nm (DLS volume distribution), PDI = 0.07, ζ-potential = −37 ± 9 mV, FT-IR = 1100 cm^−1^ (asymmetric Si-O-Si stretching), 960 cm^−1^ (Si-OH stretching), 787 cm^−1^ (symmetric Si-O-Si stretching).

### 2.3 Synthesis of CPX ⊂ SiO_2_

Silica nanoparticles loaded with CPX were synthesised following the same procedure. A solution of CPX (40 mg, 0.12 mmol, 0.012 eq), EtOH (30 mL), NH_4_OH (1.52 mL of 28 % w/v, 0.9 M) and H_2_O (125 μL, 0.28 M) was stirred until dissolution prior to addition of TEOS (2 mL in 5 mL EtOH, d = 0.933 gml^−1^, 9 mmol, 1 eq). The resulting solution was stirred for 2 h at room temperature, followed by 15 min sonication (200 W, 20 kHz), then another 1 h of stirring. The white suspension was then centrifuged (7450 rpm, 15 min) and the supernatant was removed. Resulting nanoparticles were then washed with H_2_O (3 x 25 mL), dried under vacuum and isolated as a white powder (0.75 g); diameter 127 ± 36 nm (DLS volume distribution), PDI = 0.13, ζ-potential = −28 ± 5 mV, FT-IR = 1091 cm^−1^ (asymmetric Si-O-Si stretching), 950 cm^−1^ (Si-OH stretching), 790 cm^−1^ (symmetric Si-O-Si stretching).

### 2.4 Synthesis of m-SiO_2_ and CPX@m-SiO_2_

Following the method of MCM-41 synthesis, **m-SiO_2_**_,_ were synthesised [37]. A solution of cetyltrimethylammonium bromide (CTAB) (500 mg, 1.4 mmol, 0.13 eq) was stirred at 40°C in MilliQ water (240 mL) for 10 mins. A solution of NaOH (2 mM solution in MilliQ water, 1.75 mL) was then added, followed by increasing the temperature to 80°C and stirring at 1200 rpm, TEOS was then added at a rate of 0.25 mL min^−1^ (2.5 mL, 11 mmol, 1 eq). The solution was then stirred for 2 h at 80°C. The white suspension was then centrifuged (7450 rpm, 15 min) and the supernatant was removed. Resulting nanoparticles were then washed with H_2_O (2 x 25 mL) and EtOH (25 mL). The particles were then dried under vacuum and calcinated (650°C, 12 h) to remove remaining surfactant, yielding **m-SiO_2_** (0.56 g); The diameter 136 ± 29 nm (DLS volume distribution), PDI = 0.20, ζ-potential = −20 ± 6 mV, FT-IR = 1084 cm^−1^ (asymmetric Si-O-Si stretching), 970 cm^−1^ (Si-OH stretching), 806 cm^−1^ (symmetric Si-O-Si stretching) **m-SiO_2_** were loaded with CPX to yield **CPX@m-SiO_2_**. CPX (1.16 g, 3.5 mmol) was dissolved in water (84 mL). Calcinated **m-SiO_2_** (84 mg) were then added to the CPX solution and left to stir for 24 h at room temperature. The suspension of particles was then centrifuged, and the supernatant removed. Resulting nanoparticles were dried under vacuum giving a white powder (0.076 g). FT-IR = 1084 cm^−1^ (asymmetric Si-O-Si stretching), 970 cm^−1^ (Si-OH stretching), 806 cm^−1^ (symmetric Si-O-Si stretching).

### 2.5 Quantification of antibiotics loaded by UV–Vis

UV–Vis spectroscopy measurements of the supernatant liquids after nanoparticle centrifugation were used to estimate the loading of CPX in **CPX ⊂ SiO_2_** and **CPX@m-SiO_2_** in comparison with the concentration of CPX added in the synthesis (ε = 13897 M^−1^cm^−1^, λ = 325 nm, CPX at pH = 10).

The weight percent (wt %) of antibiotic encapsulated in nanoparticles can be calculated:

$$\mathrm{encapsulatedantibiotic}\%wt=\frac{\left(\frac{\mathrm{totalantibioticadded}-freeantibiotic}{\mathrm{totalantibioticadded}}\right)\times totalantibioticadded}{\mathrm{massofnanoparticlessynthesised}}$$

### 2.6 Antibiotic release studies

Release of CPX from **CPX ⊂ SiO_2_** and **CPX@m-SiO_2_** was assessed using 5 mg/mL suspensions of particles in 10 mL water at room temperature in 50 mL falcon tubes, with applied ultrasound at varying tip displacement from the sonotrode, 40 µm (1 W), 80 µm (3 W), 120 µm (7 W), 149 µm (12 W), and 152 μm (17 W) for 5 min sonication, with a 25 % duty cycle. The sonotrode was submerged in solution at 3 cm depth, 1 cm was left between the bottom of the vessel and the tip of the sonotrode. This was kept constant to ensure cavitation characteristics were not influenced by variability in immersion depth [38]. After ultrasound was applied, the solutions were centrifuged to remove nanoparticles, and the supernatants were analysed for the quantity of CPX released. Silent release studies were carried out in the same conditions but without ultrasound at room temperature and with incremental temperatures (+4 °C, +7 °C, +9 °C, +11 °C). Absorption of CPX in solution was then measured by UV–Vis spectroscopy. The ultrasound experiments were repeated 3 times. The Beer Lambert Law was used to calculate quantities of CPX released (ε = 9949 M^−1^cm^−1^, λ = 317, CPX in water (pH = 7)).

### 2.7 LDV measurements

Sonotrode tip displacement measurements were carried out using a 1D LDV (OFV303, Polytec, Germany) at a range of power settings (1, 2, 3, 5, 7, 9, 12, 14, 17 W). The laser was focused perpendicularly to the sonotrode tip at room temperature in air. The sonotrode was driven in continuous-wave mode using its commercial driving system, and the tip velocity was recorded for 50 ms at each power level. The data was processed in MATLAB (MathWorks, USA) to determine the tip displacement amplitude. All reported tip displacement amplitudes are peak-to-peak. The standard deviation is included in graph in Fig. 4. Whilst tip displacement of a sonotrode can be variable, the short sonication durations used in this study limit the variability in observed results. All high-speed imaging was recorded at 80 kfps which is sufficient to track vibrational amplitude throughout the sonication and no significant variations in tip-vibration amplitude were observed during sonication.

### 2.8 swPCD measurements

Further acoustic detection of cavitation emissions was undertaken with a bespoke, in-house fabricated, shockwave passive cavitation detector (swPCD). The active element was 110 μm thick polyvinylidene fluoride (PVdF), designed for high-sensitivity to bubble-collapse shockwaves [35]. The swPCD has an active element diameter of 15 mm. A PicoScope (PicoScope 3000 series, Pico Technology, UK) was used to record the voltage output from swPCD, and acoustic spectra generated via FFT during sonication of different samples including water, **m-SiO_2_**, **SiO_2_**, **CPX ⊂ SiO_2_**, and **CPX@m-SiO_2_**. Nanoparticle samples were made in concentrations of 1 mg/mL in water. The swPCD was submerged in the samples at room temperature in a tank of size 9 cm × 5 cm × 4 cm along with the sonotrode. The sonotrode was submerged tip downwards at 3 cm depth, 1 cm was left between the bottom of the tank and the sonotrode. The swPCD was fully submerged in solution and clamped orthogonally to the sonotrode, at a distance on 7 cm. The acoustic spectra taken for a range of tip displacements (40 μm (1 W), 80 μm (3 W), 120 μm (7 W), 152 μm (17 W)). The swPCD was connected to PicoScope 5000 series (Pico Technologies, UK) for data collection at 10 × 10^6^ samples/s. Acoustic emissions were recorded for a total duration of 100 ms, triggered approximately 2 s Into the sonication with a total of 12 data sets per variable. Acoustic reflections are likely to be present given the wavelength of the ultrasound at 20 kHz. However, the primary emissions being detected are that from the cavitation cloud generated directly at the sonotrode tip, which are far more intense than that of any potentially reflected fundamental waves. Short sonication durations were employed throughout this study to best mitigate large temperature variations, and temperature levels were monitored before and after sonication. Data was collected using PicoScope 6 software and exported as a.Mat file for processing the data on MATLAB.

### 2.9 Capillary experiment setup

The full capillary experimental arrangement is described in *Song et al* [39]. In summary, samples of each particle were observed to cavitate in a 500 µm polycarbonate capillary (Paradigm Optics, Vancouver, WA, USA) with ultrasound provided by 2 s sonication of a 20 kHz sonotrode (Fisherbrand Model 120 Sonic Dismembrator, 3 mm probe diameter) at tip displacements of 50, 60 and 70 μm. The sonotrode was mounted vertically, from the top of the tank, perpendicular to the capillary at a distance of 1 mm. The capillary is enclosed within a custom-made tank measuring 420 × 438 × 220 mm^3^ filled with degassed, deionised water. A high-speed camera (Photron Fastcam SA-Z, Photron, Bucks) imaging at 80,000 frames per second (fps) over the entire sonication duration captured the cavitation response of the particles in the capillary at high temporal resolution and magnification (5 x 0.14 Numerical Aperture (NA), focal length (in air): 40.0 mm, Mitutoyo, Kawasaki Japan). Illumination was provided by a synchronous (to frame capture) 10-ns pulsed laser coupled to a liquid light guide and collimator lens (CAVILUX Smart, Cavitar Finland).

## 3 Results and discussion

### 3.1 Particle formation and characterisation

The non-porous silica nanoparticles with encapsulated antibiotic, **CPX ⊂ SiO_2,_** were made with addition of the antibiotic during the synthesis as described in materials and methods. The properties were compared with mesoporous nanoparticles where the antibiotic is adsorbed in the porous network, **CPX@m-SiO_2_** (Fig. 1).

**Fig. 2:**
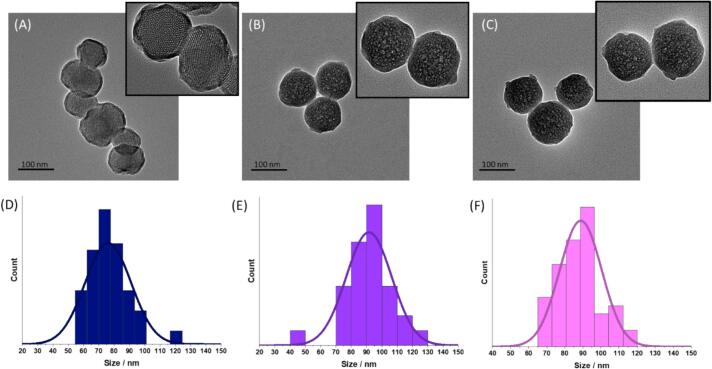
TEM images of (A) m-SiO_2_, (B) SiO_2_, and (C) CPX ⊂ SiO_2_along with corresponding size distribution histograms of (D) m-SiO_2_, (E) SiO_2_, and (F) CPX ⊂ SiO_2_ (n = 50).

Characterisation of particle sizes was carried out using dynamic light scattering (DLS) and transmission electron microscopy (TEM). DLS data shows an average diameter measured by volume as 124 ± 35 (PDI = 0.07), 127 ± 36 nm (PDI = 0.13), and 136 ± 29 nm (PDI = 0.20), for **SiO_2,_ CPX ⊂ SiO_2_**, and **m-SiO_2_**, respectively (Table S1, Fig. S1, Supporting Information). TEM images confirmed good monodispersity of particles, sizes were determined to be 70 ± 10 nm, 88 ± 12 nm, and 77 ± 12 nm for **SiO_2,_ CPX ⊂ SiO_2_**, and **m-SiO_2_**, respectively, confirming similar sizes for all particles within the standard deviation (Fig. 2). The smaller sizes from TEM compared with DLS are expected for silica particles due to the hydrodynamic radius measured by DLS. The TEM images of the nanoparticles also confirmed the morphology, where the hexagonal porous structure of **m-SiO_2_** could be seen and the pore sizes were measured to be 2.7 ± 0.6 nm (Fig. 2A). For **SiO_2_** and **CPX ⊂ SiO_2_** the morphology was the same between the two samples and a clear porous structure, like that of **m-SiO_2_**, was not observed (Fig. 2B,C). FT-IR was used to characterise the materials, and the Si-O-Si and Si-O stretching bands confirmed the hydrolysis and condensation of Si-O in the nanoparticle structure (Fig. S2, Supporting Information).

**Fig. 3:**
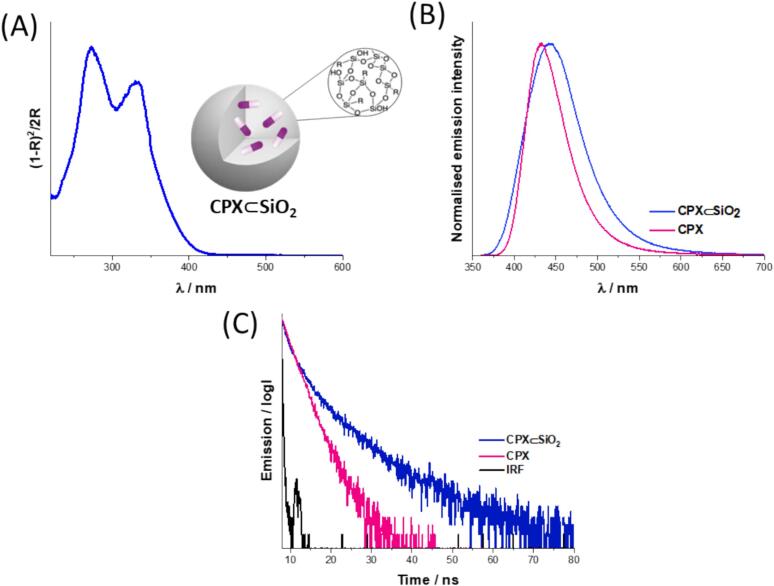
Composition characterisation of CPX ⊂ SiO_2_ in powder form by identification of the presence of CPX as compared with CPX by (A) UV-Vis spectroscopy, (B) fluorescence spectroscopy (λ_exc_ = 330 nm, λ_max_ = 443 nm) and (C) luminescence lifetime spectroscopy (λ = 375 nm).

The loading of CPX within **CPX ⊂ SiO_2_** and **CPX@m-SiO_2_** was estimated from the analysis of the remaining CPX in the supernatant after centrifugation by UV–Vis spectroscopy (methods 2.5). The CPX loading was calculated to be 4 % wt for **CPX ⊂ SiO_2_**, whilst for **CPX@m-SiO_2_** it was found to be 10 % wt. The loading of CPX is expected to be higher in the mesoporous particles, **CPX@m-SiO_2_** due to the large surface area for drug adsorption in the channel network of the silica structure, whereas in **CPX ⊂ SiO_2_** the CPX encapsulation is based on interactions during the formation of the silica network. It is important to note that we found that the encapsulation efficiency of CPX in **CPX ⊂ SiO_2_** is sensitive to its overall concentration as we tested variations of solvent additions and our reported method has been optimised. The presence of CPX in **CPX ⊂ SiO_2_** and **CPX@m-SiO_2_** was further confirmed by solid state UV–Vis spectroscopy of the nanoparticle powders. Absorbance bands at λ = 268 nm and 328 nm were observed for the nanoparticle powders (Fig. 3, Fig. S3, Supporting Information) and attributed to π-π* transitions of CPX, correlating well with aqueous solution of the antibiotic in basic conditions (pH = 10) (Fig. S4, Supporting Information) [[40], [41]].

CPX is fluorescent and its properties in the silica nanoparticles were studied using fluorescence and luminescence lifetime studies. The fluorescence signal of CPX as a powder exhibited a bathochromic shift of 10 nm when encapsulated in the silica nanoparticle (**CPX ⊂ SiO_2_**) compared to powders of free CPX, whilst **CPX@m-SiO_2_** showed the same λ_max_ (λ_max_ = 433 nm) as the CPX powder (Fig. S3, Supporting Information). The differences in emission maxima for **CPX ⊂ SiO_2_** and **CPX@m-SiO_2_** is attributed to the different forms, anionic versus zwitterionic, of CPX which was observed in the solid-state UV–Vis. The luminescence lifetime of CPX shows biexponential decay in the solid state, with two components with similar values (τ_1_ = 2.4 (75 %), τ_2_ = 4.6 (25 %)). The luminescence lifetime of CPX when encapsulated in silica, **CPX ⊂ SiO_2_**, was found to overall increase compared with CPX powder, τ_1_ = 2.0 (25 %), τ_2_ = 6.3 (75 %) with the long component from τ_2_ = 4.6 (25 %) to τ_2_ = 6.3 (75 %) and where the short component is significantly reduced in amplitude from 75 % to 25 % (Fig. 3C). The same behaviour was observed for **CPX@m-SiO_2_** (Fig. S3, Supporting Information) giving τ_1_ = 1.7 (60 %), τ_2_ = 6.3 (40 %). The increase in lifetime can be attributed to the encapsulation of CPX in the silica matrix due to reduced rotational mobility [42]. The presence of the two lifetime decay components in the solid state may indicate the presence of two forms of CPX (zwitterionic versus anionic) or hydrogen bonding involvement within the CPX and/or the silica network.

### 3.2 Characterisation of tip displacement of 20 kHz sonotrode

**Fig. 4:**
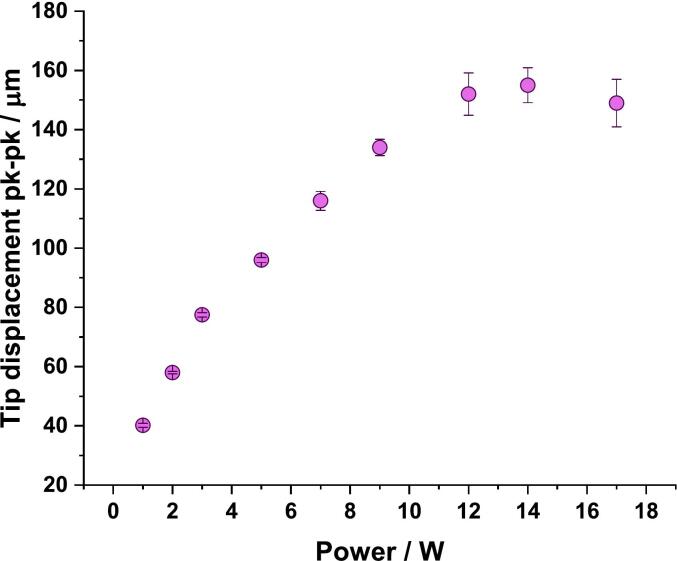
1D LDV measurement of a sonotrode demonstrating the relationship between power setting and displacement at the tip.

1D LDV measurements were used to determine the displacement amplitude at the tip of the sonotrode. LDV is a technique commonly used to characterise the vibrational motion of ultrasonic devices [[43], [44], [45]]. Here, the LDV beam is focussed on the tip of the sonotrode. The Doppler shift of the reflected signal is used to characterise the normal-to-surface vibration velocity, from which displacement is derived [[46], [47]]. The sonotrode exhibited a linear relationship between tip displacement amplitude and input power up to 12 W (Fig. 4). However, at higher input powers (14 W and 17 W), nonlinear effects become significant, leading to a reduction in displacement at the highest input power, where there may be loss of energy due to the age of the device resulting in wear which effects the efficiency of operation and has led to the harmonics increasing with respect to voltage. This reduces tip displacement in such a non-linear regime of operation. Consequently, at the highest power levels, the system entered a nonlinear regime, where increased mechanical losses and reduced power transfer efficiency, along with the excitation of parasitic modes such as harmonics, contributed to a decrease in tip displacement.

### 3.3 Controlled drug release using 20 kHz sonotrode

**Fig. 5:**
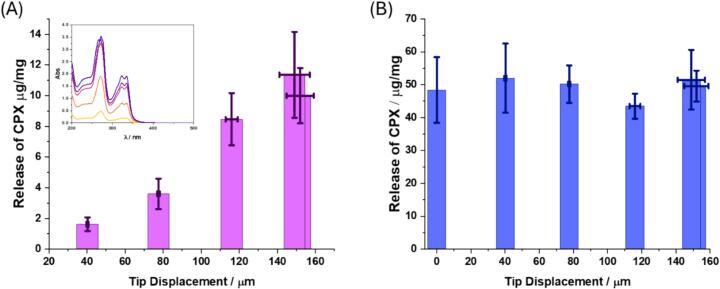
Release of CPX from dispersions of nanoparticles (A) CPX ⊂ SiO_2_ (insert UV–Vis spectra of released CPX) and (B) CPX@mSiO_2_ with increasing tip displacement using a 20 kHz sonotrode (n = 3).

Drug release from dispersion of **CPX ⊂ SiO_2_** (5 mg/mL) was monitored after application of 20 kHz ultrasound for 5 min; the nanoparticle dispersion was subjected to centrifugation, and the supernatant was characterised using UV–Vis spectroscopy to calculate the amount of CPX released from **CPX ⊂ SiO_2_** (Fig. 5 A). The calculated release of CPX ranged from 1.7 ± 0.3 (40 μm, 2 W) to 8.5 ± 1 μg/mg (120 μm, 8 W) showing a linear increase in release with tip displacement (Fig. 5A, Fig. S5, Supporting Information), suggesting drug release is correlated with the tip displacement amplitude of the sonotrode. The CPX % release was also calculated to range from 3 ± 0.5 to 25 ± 5.4 % of the total loading of the **CPX** in **CPX ⊂ SiO_2_** (Fig. S5, Supporting Information). The large error involved in amplitudes greater than 120 μm is due to the uncertainty in the values of tip displacement, reflecting the LDV measurements (Fig. 4). There was minimal release of CPX under static conditions at room temperature (0.7 ± 0.2 μg/mg). These results demonstrate that the release from **CPX ⊂ SiO_2_** is caused primarily by cavitation induced effects upon application of ultrasound, suggesting that **SiO_2_** is a cavitation sensitive drug delivery vehicle. Drug release from **CPX ⊂ SiO_2_** was also compared with CPX loaded **m-SiO_2_** (**CPX@m-SiO_2_**). The same amount of drug release was observed for **CPX@m-SiO_2_** with and without ultrasound and there was no dependence on tip displacement (Fig. 5B). Although the amount of CPX released was higher (∼50 μg/mg) and had a greater release of CPX as a function of total particle loading (∼50 %) (Fig. S5, Supporting Information) for **CPX@m-SiO_2_** than **CPX ⊂ SiO_2_**, it is not controlled, and observed in both static (silent) and ultrasound conditions. Off-target effects and over-use of CPX has been linked to adverse side effects such as renal failure and antibiotic resistance [[48], [49]], therefore controlled and triggered drug delivery is paramount to the efficacy of future antibacterial treatments.

The amount of CPX released from **CPX ⊂ SiO_2_** even at 40 μm tip displacement is clinically relevant (5.7 mg/L drug release) against bacterial strains such as *E. coli*, *Pseudomonas aeruginosa,* and *Staphylococcus aureus*. For example, the minimum inhibitory concentration (MIC) of CPX against *E. coli* has been determined as 0.06 mg/L [50]. *S. aureus* and *P. aeruginosa* are classified as ESKAPE pathogens, which are the leading cause of nosocomial infections, the majority of which are multidrug resistant, they are also present in acute and chronic wounds [51]. The clinical dose for a local treatment of CPX for eye infections is estimated to be 3 mg/ml (prescribed in drops) [52]. The synergistic effects between antibiotic activity and ultrasound have also been found to enhance antibiotic effects due to selective action at the site of infection, which could enable lower dosages required [53]. Our results show greater drug release quantities compared with previously reported CPX loaded chitosan nanoparticles which demonstrated 1.5–6.5 mg/L release [54]. Other studies have also noted an increase in drug release with increasing applied power of ultrasound from materials such as micelle structures and liposomes which are known to break apart due to cavitation, but it has not been demonstrated for non-porous silica nanoparticles [[3], [5], [55]].

The drug release effects observed can be attributed to cavitation related mechanisms since the silica structure is not broken down by ultrasound due to strong Si-O covalent bonds [[27], [56]]. Cavitating bubbles in solution with drug delivery vehicles promote several different phenomena including most notably mechanical effects, chemical effects, and thermal effects [57]. Shockwaves and microjets are classified as mechanical effects and occur due to inertial bubble oscillations and collapse [58]. These can promote shear stresses on surrounding materials which can in turn promote drug release [57]. Chemical effects arise from the production of free radicals in solution, such as reactive oxygen species (ROS) [59]. Temperature increase in the solution during the sonication is observed, which is primarily due to frictional effects from tip-oscillation with water. Highly localised and transient thermal effects occurring from collapsing cavitation bubbles and non-linear acoustic radiation [[60], [61]] may contribute to heating.

A duty cycle of 25 % was used during application of ultrasound. This was chosen as under continuous ultrasound application the solution reaches high temperatures up to 48°C, making it incompatible for biological systems. A 25 % duty cycle is the lowest duty cycle programmable on the acoustic device. We independently checked the temperature change during ultrasound conditions with 120 μm tip displacement at 25 % duty cycle to evaluate the contribution of thermal effects to drug release (Fig. S6, Supporting Information). To estimate the release of CPX from the **CPX ⊂ SiO_2_** under silent conditions, the particles were treated at varying temperatures to correspond to the increase (+4 °C, + 7°C, + 9°C, + 11°C), observed under ultrasound conditions. The drug release was found to have no significant increase with temperature variation with only 1.7–2 μg/mg of CPX (5.5–6 wt% release) which lower than ultrasound conditions (8.5 ± 1 μg/mg). These results indicate that thermal contribution during sonication is not the dominant effect for the drug release. The drug release is attributed to mechanical effects of the cavitating bubbles in solution, leading to disruption of the structure of the particles. It is important to note that it has been found that Reactive Oxygen Species production has no effect on the silica framework of the nanoparticles [62].

### 3.4 Cavitation response of nanoparticles

To evaluate the effects of cavitation on the particles in solution, cavitation activity was evaluated in water along with nanoparticle suspensions of a concentration of 1 mg/mL. A swPCD was used in a water tank (Fig. S7 Supporting Information), similar to previously reported detectors [[35], [63]]. A swPCD was chosen rather than a hydrophone for this experiment due to the small volume of solution used in the experimental set up. Hydrophones are not suitable to use where there is close proximity to cavitation activity, the swPCD is designed for sensitivity and tolerance to bubble collapse shockwaves which are typically observed during inertial cavitation [64]. For a given driving frequency (*ƒ_0_*), sub-harmonic (*ƒ_0_*/2), ultra-subharmonics ((2*n* + 1)*ƒ_0_*/2) and background noise are observed in the measured acoustic spectra when inertial cavitation is present in solution [[64], [65], [66]]. These spectral features are generally exclusively attributed to cavitation activity, allowing their presence and intensity to be analysed [[67], [68]]. To examine the effect of the porosity of the nanoparticles on the cavitation response, two silica nanoparticle structures were investigated: non-porous silica nanoparticles (**SiO_2_** and **CPX ⊂ SiO_2_**) and mesoporous silica nanoparticles (**m-SiO_2_** and **CPX@m-SiO_2_**). The spectral features of the cavitation activity measurements adding these samples were examined, using deionised water as a control.

The sub-harmonic signal observed at *ƒ_0_*/2 is due to periodic shock waves caused by bubbles collapsing and is frequently used to analyse acoustic spectra [[69], [70]]. Higher-order subharmonics, including *ƒ_0_*/3, *ƒ_0_*/4, and *ƒ_0_*/5 are also characteristic of cavitation, and typically appear at higher driving amplitudes [71], increasing incrementally through these subharmonic orders as driving amplitude increases. The peaks *ƒ_0_*/2 and *ƒ_0_*/5 were used as an indicator of cavitation occurring in solution for the aforementioned samples.

**Fig. 6:**
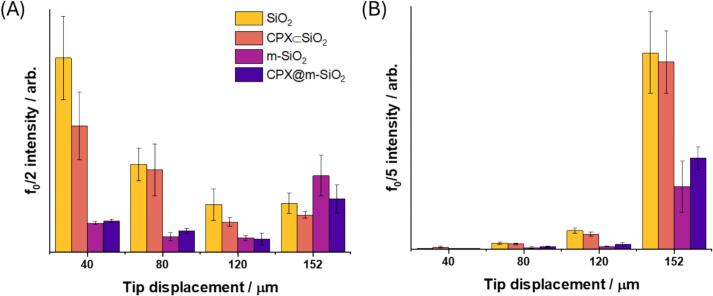
Spectral peak amplitudes for (A) *ƒ_0_*/2 and(B) *ƒ_0_/*5 measured using a swPCD in the presence of water, SiO_2_, CPX ⊂ SiO_2_, m-SiO_2_ and CPX@m-SiO_2_ (n = 12).

In water, where the ultrasound field was excited by 40 μm sonotrode tip displacement (1 W), *ƒ_0_*/2 was observed in the acoustic spectrum measurement, as well as ultra-subharmonics. However, higher-order sub-harmonics were not seen (Fig. S8, Supporting information). With increasing tip displacement, *ƒ_0_*/2 intensity decreased, and higher-order sub-harmonics and acoustic noise increased (Fig. 6). Such behaviour has been reported previously, where increasing the displacement amplitude of acoustic devices can lead to an increase in intensity of higher order sub-harmonics [71]. The acoustic spectra of the particles in solution were then analysed with respect to water.

For **SiO_2_** the intensity of *ƒ_0_*/2 at 40 μm (1 W) tip displacement was higher than that of water, and at higher tip displacements acoustic noise and sub-harmonics remained at a higher intensity than for water (Fig. 6A, Fig. S9 Supporting Information). A decrease in *ƒ_0_*/2 intensity corresponding with an increase in *ƒ_0_*/5 intensity was observed with increasing amplitude like that of water, which was also seen for **CPX ⊂ SiO_2_** (Fig. S10 Supporting Information). The intensities of the sub-harmonics were within the standard error of **SiO_2_**, suggesting the drug loading does not affect the cavitation behaviour of **SiO_2_** (Fig. 6). Monitoring of the subharmonic response gives an indication of the cavitation present. Studies have shown that, with increasing driving amplitude, the subharmonic response will transition through *ƒ_0_*/*m* subharmonics, with *m* increasing through integer values [63]. Effectively, the period between the cavitation cloud collapses increases with higher driving amplitudes, as the cavitation cloud grows larger between collapses. Hence, in Fig. 6 we observe higher f0/2 subharmonics at lower tip displacement amplitudes, with this subharmonic integer increasing up to *ƒ_0_*/5 at higher tip displacement amplitudes. The observed difference in SiO_2_ and m-SiO_2_ subharmonic signatures relates to the prevalence of higher order cavitation in the subharmonic acoustic spectra. Here, the m-SiO2 exhibits less f0/5 subharmonic collapses at higher tip displacement amplitudes, rather it is producing more *ƒ_0_*/2 (along with *ƒ_0_*/3 and *ƒ_0_*/4) subharmonic collapses due to cavitating less intensely and at a lower order subharmonic, generally. This is in agreement with our observed cavitation in the capillary (Fig. 8), which indicates that the m-SiO_2_ particles require greater tip displacement to begin cavitating and hence will require greater tip displacement to transition to higher order subharmonics.

**Fig. 7:**
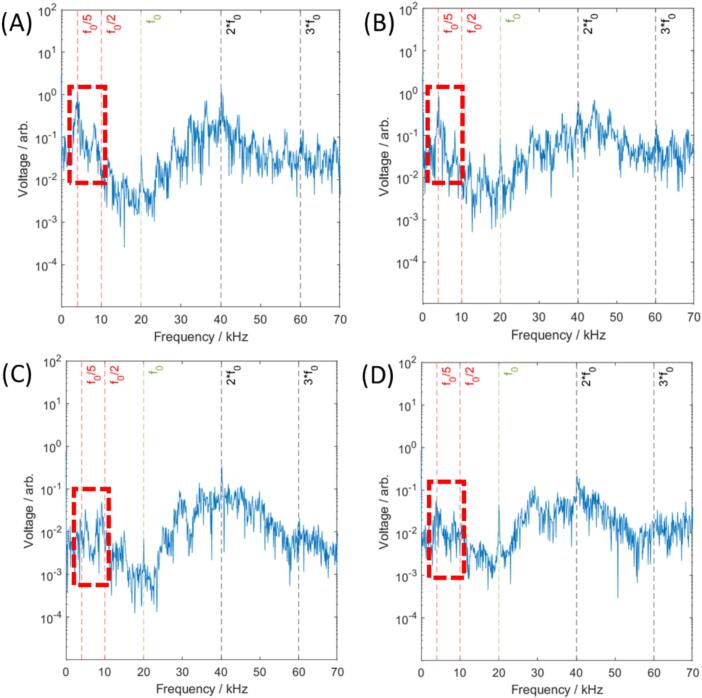
Acoustic spectra from 20 kHz sonotrode at 152 μm (17 W) tip displacement sonicating an aqueous dispersion of (A) SiO_2_ (B) CPX ⊂ SiO_2_ (C) m-SiO_2_ and (D) CPX@m-SiO_2_.

A decrease in *ƒ_0_*/2 intensity corresponding with an increase in *ƒ_0_*/5 intensity was also observed for **m-SiO_2_** (Fig. 6, Fig. S11 Supporting Information) and **CPX@m-SiO_2_** (Fig. 6, Fig. S12 Supporting Information) at increasing sonotrode tip displacement amplitudes. The amplitude of *ƒ_0_*/2 at 40 μm (1 W) tip displacement was similar to that of water and thus significantly less than observed for **CPX ⊂ SiO_2_** and **SiO_2_** (Fig. 6A, Fig. S8 Supporting Information). At higher tip displacement amplitude (152 μm, 17 W), *ƒ_0_*/5 amplitude increased, but to a significantly lower amplitude than observed for **CPX ⊂ SiO_2_** and **SiO_2_** (Fig. 6B, Fig. 7). The difference between the acoustic spectra of **m-SiO_2_** (Fig. S11 Supporting Information) and **CPX@m-SiO_2_** (Fig. S12 Supporting Information) was not significant, which correlates with the immediate release of CPX from **CPX@m-SiO_2_** in solution, resulting in **m-SiO_2_** and **CPX@m-SiO_2_** having a similar porous structure in water. The trends in acoustic spectra suggest that non-porous SiO_2_ is a cavitation agent, promoting cavitation in solution, more so than porous SiO_2_.

**Fig. 8:**
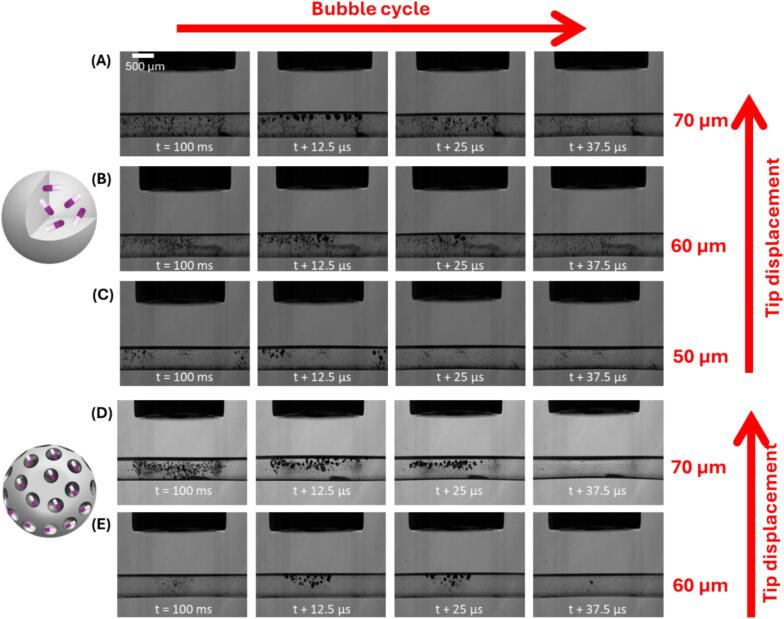
Representative Images taken from the capillary high-speed imaging sequences during capillary experiments recorded at 80 kfps such that each row presents one acoustic cycle from a 20 kHz US transducer with aqueous dispersions of CPX ⊂ SiO_2_ at (A) 70 μm (B) 60 μm and (C) 50 μm and CPX@m-SiO_2_ at (D) 70 μm (E) 60 μm.

To further confirm the use of **CPX ⊂ SiO_2_** and **SiO_2_** as cavitation agents compared with **m-SiO_2_** and **CPX@m-SiO_2_**, capillary experiments were carried out with degassed water using high-speed imaging with particle concentrations of 5 mg/mL. In degassed solution alone, the sonotrode excitation does not result in cavitation. No cavitation was observed for any samples at 40 μm tip displacement, **CPX ⊂ SiO_2_** (Fig. 8A, B, C) and **SiO_2_** (Fig. S13 Supporting Information) produced cavitation at 50 μm tip displacement, whilst for **m-SiO_2_** (Fig. S13 Supporting information) and **CPX@m-SiO_2_** (Fig. 8D, E) cavitation initiated at 60 μm. The higher sonotrode displacement amplitude required to produce cavitation in the presence of the porous samples correlates with the swPCD data, suggesting that **CPX ⊂ SiO_2_** and **SiO_2_** are superior cavitation agents compared with **m-SiO_2_** and **CPX@m-SiO_2_**.

There are many factors that can affect the cavitation phenomena observed by the drug loaded particles. The amorphous particles with encapsulated drug show a rougher surface by TEM (Fig. 2) and a variation of voids, caused by the drug encapsulation process and/or formation of the silica network during the hydrolysis process. This allows for more nucleation sites on the surface particles which leads to lower amplitude thresholds. The m-SiO_2_ particles present a smoother outer surface with channels. The amount of trapped gas in the particles may also play an effect in the cavitation process. The gas trapped in the amorphous silica particles during their formation process and for the ones with encapsulated drug may be significant in comparison to the m-SiO_2_ particles which have an organised large porous network (average pore size 2.7 nm) where adsorbed gas or drug can leak uncontrollably. Finally, the process of drug release is different as in the amorphous particles. In amorphous silica we hypothesize that cavitation affects the vibration/breathing modes of the Si-OH bonds and the hydrogen bonded network in the surface of silica which leads to drug release.

It is important to note that during cavitation, the small pores of the m-SiO_2_ particles are subject to capillary forces that allow the penetration of surrounding water into the pores, facilitating the release of CPX into the bulk liquid[[72], [73]]. The addition of acoustic streaming in the bulk liquid and microstreaming around the particles adds forces across all the pores across the surface of the m-SiO_2_ particles, which could contribute to the leaking of the CPX [74]. The process of CPX release is different in the amorphous particles for which we attribute the controlled drug release to the opening of voids in the silica framework upon ultrasound rather than simply displacement of CPX from the adsorbed silica network.

## 4 Conclusions

This work describes a novel approach to antibiotic delivery using cavitation to trigger release from non-porous silica nanoparticles. A significant release of antibiotic in the presence of low frequency ultrasound was observed, correlating with an increase in displacement amplitude of the sonotrode. High-speed imaging and shock wave passive cavitation detection (swPCD) also elucidated an enhanced cavitation response when non-porous silica nanoparticles were present. Such delivery enhancement was not observed with mesoporous silica nanoparticles which showed uncontrolled antibiotic release. The amount of antibiotic release is suited for clinically relevant antibiotic treatment. These results open up the design of silica nanoparticles as sensitive vehicles for drug delivery and as promising new agents for translation to clinical applications due to their biocompatibility.

## CRediT authorship contribution statement

**Grace Ball:** Writing – original draft, Methodology, Investigation, Formal analysis, Conceptualization. **Jack Stevenson:** Writing – review & editing, Visualization, Validation, Software, Investigation, Formal analysis, Data curation. **Faraz Amini Boroujeni:** Writing – review & editing, Visualization, Validation, Software, Formal analysis, Data curation. **Ben Jacobson:** Writing – review & editing, Visualization, Validation, Software, Formal analysis, Data curation. **Sarah A. Kuehne:** Writing – review & editing, Supervision. **Margaret Lucas:** Writing – review & editing, Funding acquisition. **Anthony Damien Walmsley:** Writing – review & editing, Writing – original draft, Supervision, Funding acquisition, Conceptualization. **Paul Prentice:** Writing – review & editing, Supervision, Methodology, Conceptualization. **Zoe Pikramenou:** Writing – review & editing, Writing – original draft, Supervision, Funding acquisition, Conceptualization.

## Declaration of competing interest

The authors declare the following financial interests/personal relationships which may be considered as potential competing interests: Zoe Pikramenou reports was provided by Engineering and Physical Sciences Research Council. If there are other authors, they declare that they have no known competing financial interests or personal relationships that could have appeared to influence the work reported in this paper.
