# Supplementary material for: Non-porous silica nanoparticles as a cavitation sensitive vehicle for antibiotic delivery
Source: Ultrason Sonochem. 2025 Mar 17;116:107316. doi: 10.1016/j.ultsonch.2025.107316 (PMC11981769; doi:10.1016/j.ultsonch.2025.107316)
Supplement: Supplementary Data 1 [file mmc1.docx]

**Supporting Information**

**Non-Porous Silica Nanoparticles as a Cavitation Sensitive Vehicle for Antibiotic Delivery**

Grace Ball^a^, Jack Stevenson^b^, Faraz Amini Boroujeni^b^, Ben Jacobson^b^, Sarah A. Kuehne^c^, Margaret Lucas^b^, Anthony Damien Walmsley^d^, Paul Prentice^b^, Zoe Pikramenou^a^

*^a^School of Chemistry, University of Birmingham, Edgbaston, B15 2TT, UK.*

*^b^ Glasgow address please fill in*

*^c^School of Science & Technology, Nottingham Trent University, Nottingham, NG11 8NS, UK.*

*^d^ School of Dentistry, College of Medical and Dental Sciences, University of Birmingham, Birmingham, B5 7EG, UK.*

**Table S1.** Summary of nanoparticle sizes of **m-SiO_2_**, **SiO_2_**, and **CPX**$\boldsymbol{\subset}$**SiO_2_** Dynamic Light Scattering measurements are performed at 25 °C in MilliQ water, PDI = Polydispersity Index (n = 5). Mean size by TEM is determined based on 50 nanoparticle measurements.


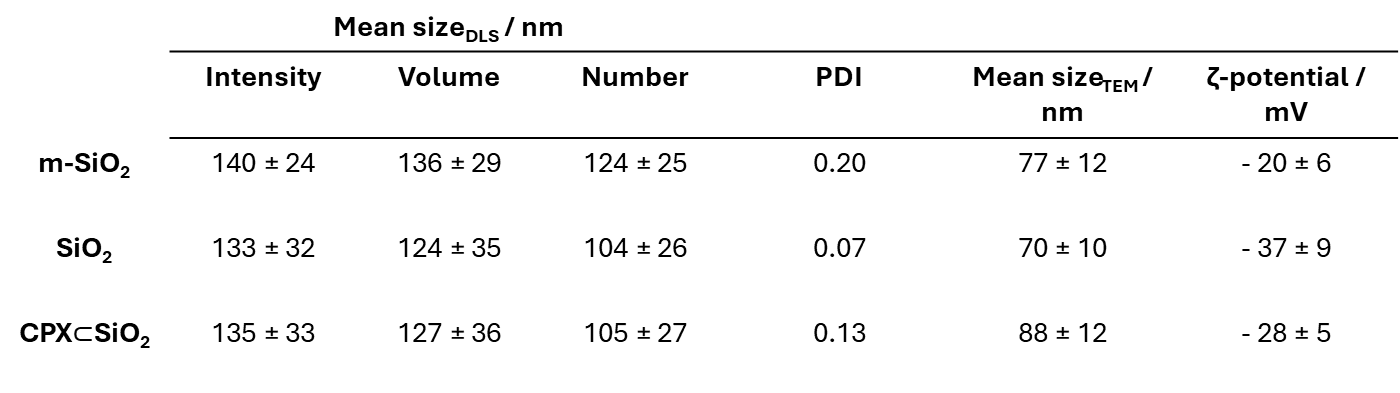


**
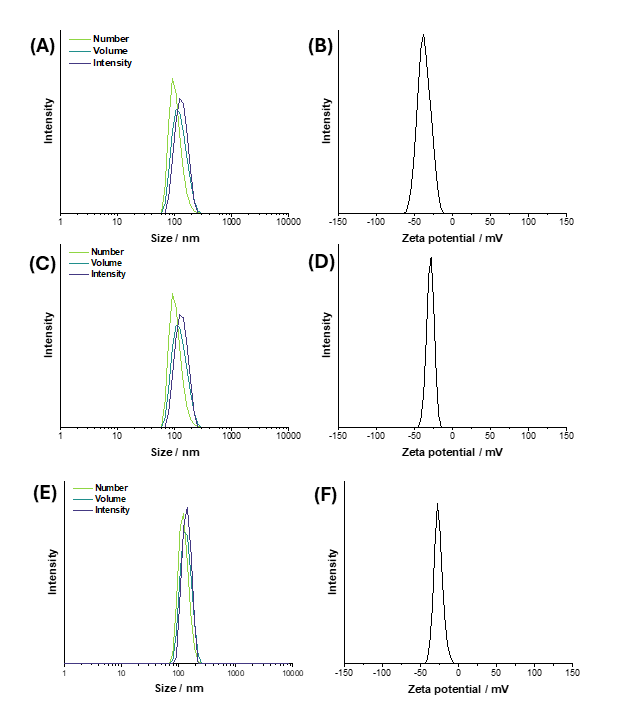
**

**Figure S1.** DLS analysis of nanoparticle sizes of (A) SiO_2_, (C) **CPX**$\boldsymbol{\subset}$**SiO_2_**, and (E) **m-SiO_2_**, and ζ – potential of (B) SiO_2_, (D) **CPX**$\boldsymbol{\subset}$**SiO_2_**, and (F) **m-SiO_2_**,


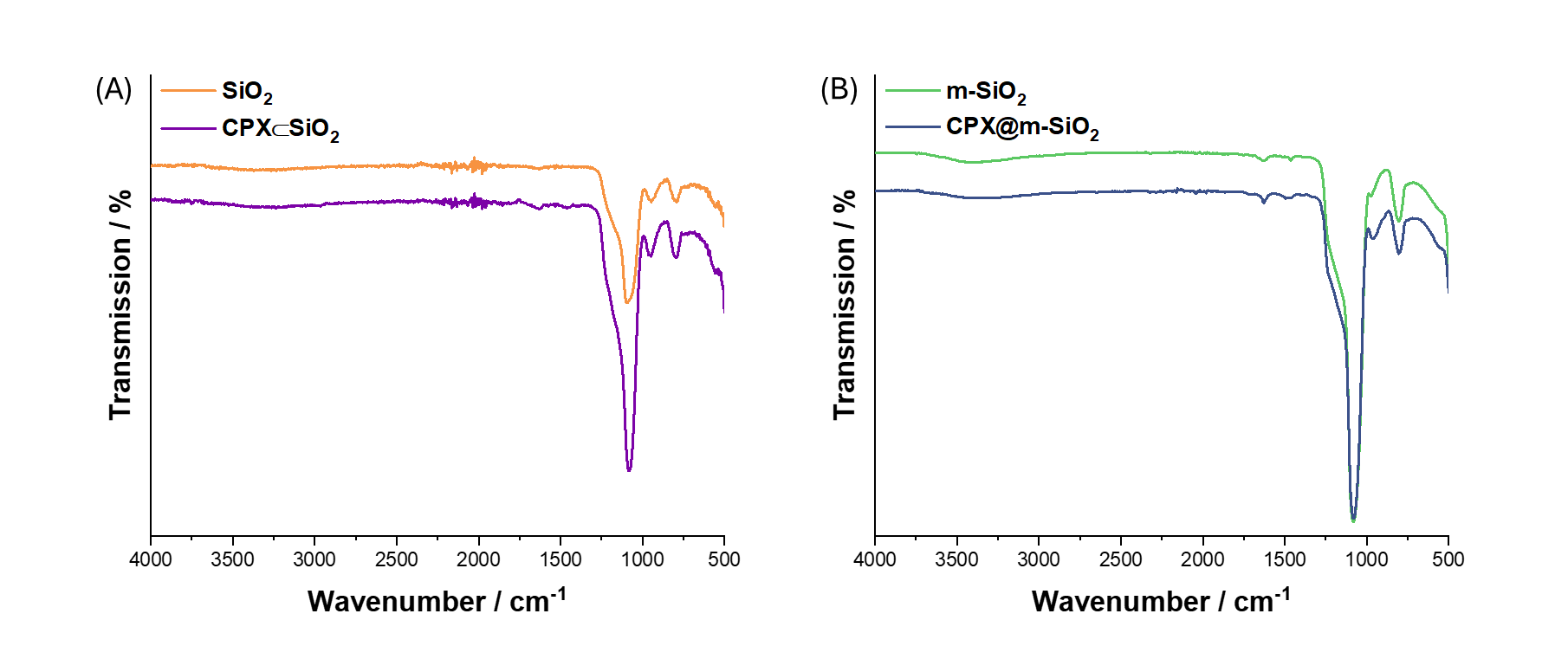


**Figure S2.** FTIR spectra of (A) **SiO_2_** and **CPX**$\boldsymbol{\subset}$**SiO_2_** and (B) **m-SiO_2_** and **CPX@m-SiO_2_**.

**
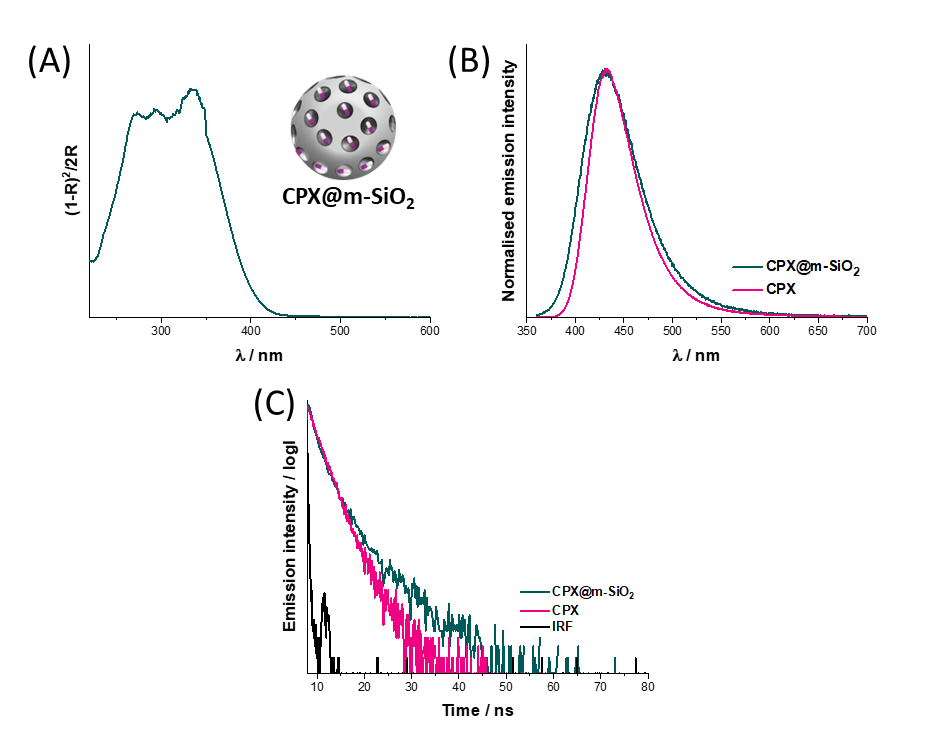
**

**Figure S3.** Composition characterisation of powders of **CPX@m-SiO_2_** by identification of the presence of CPX as compared with CPX by (A) UV-Vis spectroscopy, (B) fluorescence spectroscopy (λ_exc_ = 330 nm, λ_max_ = 443 nm)and (C) luminescence lifetime spectroscopy (λ = 375 nm).

**Figure S4.** UV-Vis spectra of CPX dissolved in EtOH/NH_4_OH (0.9 M).


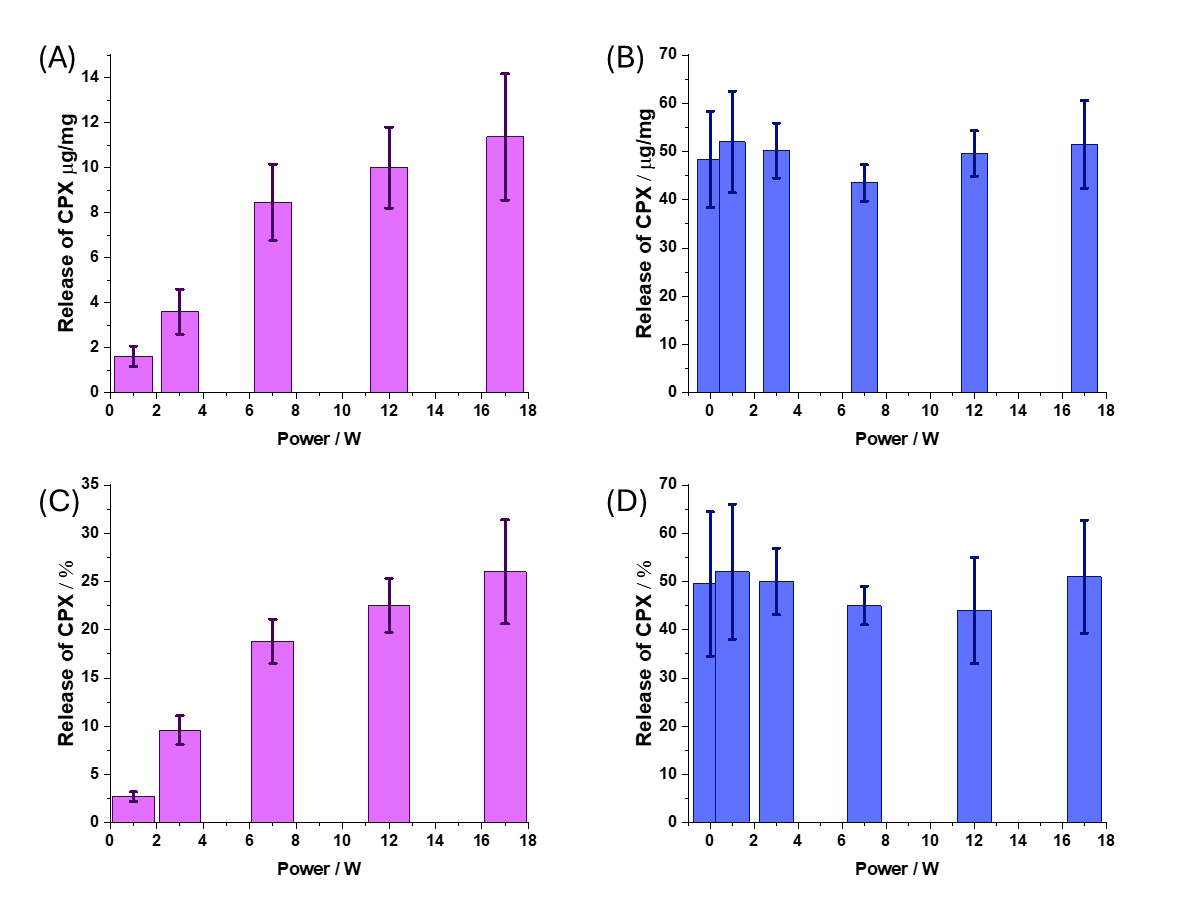


**Figure S5.** Quantitative release of CPX relevant to sonotrode power expressed per nanoparticle weight and % wt (CPX released/CPX encapsulated) (A) and (C) **CPX**$\boldsymbol{\subset}$**SiO_2_** and (B) and (D) **CPX@m-SiO_2_.**

**Figure S6:** Temperature change observed for **CPX**$\boldsymbol{\subset}$**SiO_2_** dispersed in solution at 120 μm tip displacement over a 25 % duty cycle with 5 minutes applied ultrasound.


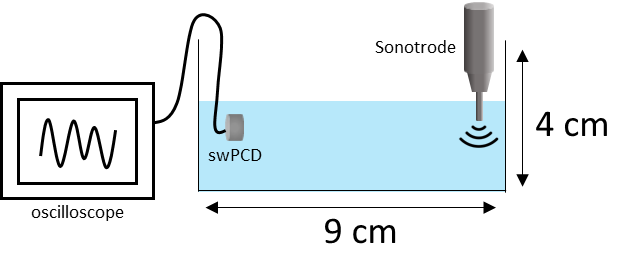


**Figure S7**. Schematic of swPCD measurements of the sonotrode in water in a tank size of 9 cm × 5 cm × 4 cm (l,b,h).


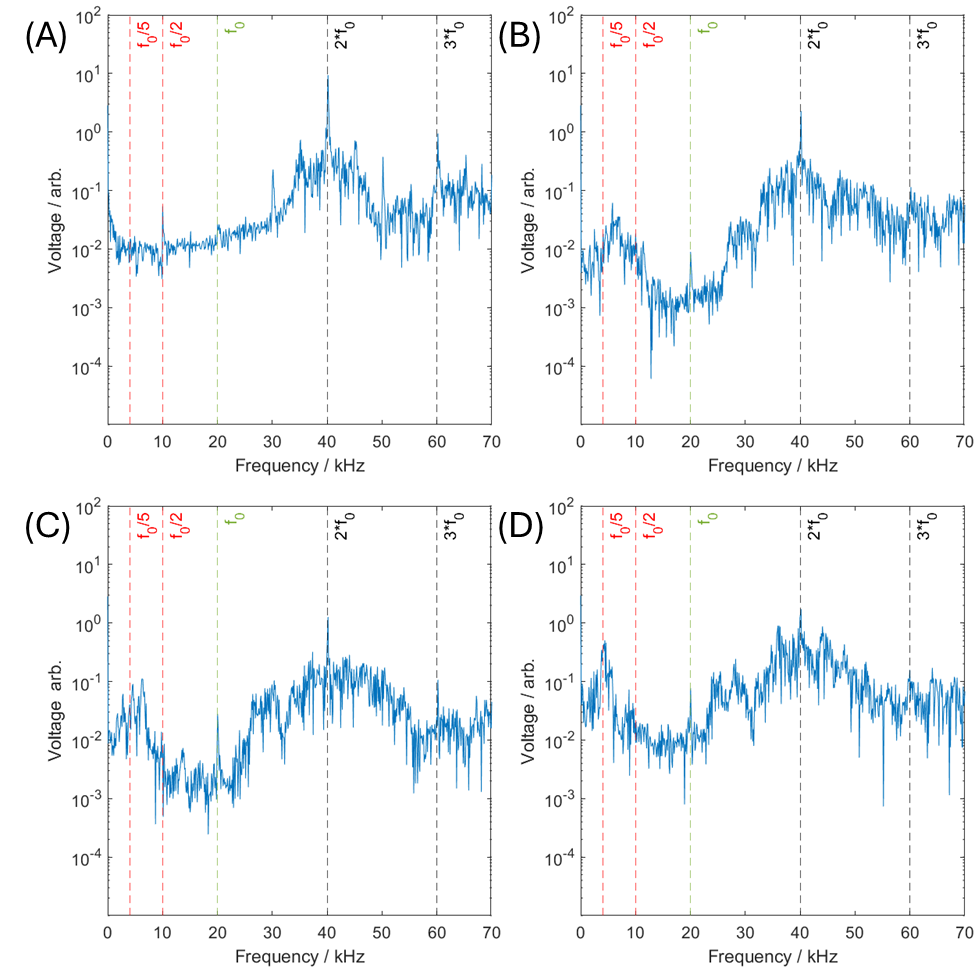


**Figure S8**. FFT of acoustic spectra taken with a swPCD of water with applied ultrasound at tip displacement amplitudes (A) 40 μm (B) 80 μm (C) 120 μm and (D) 152 μm

**
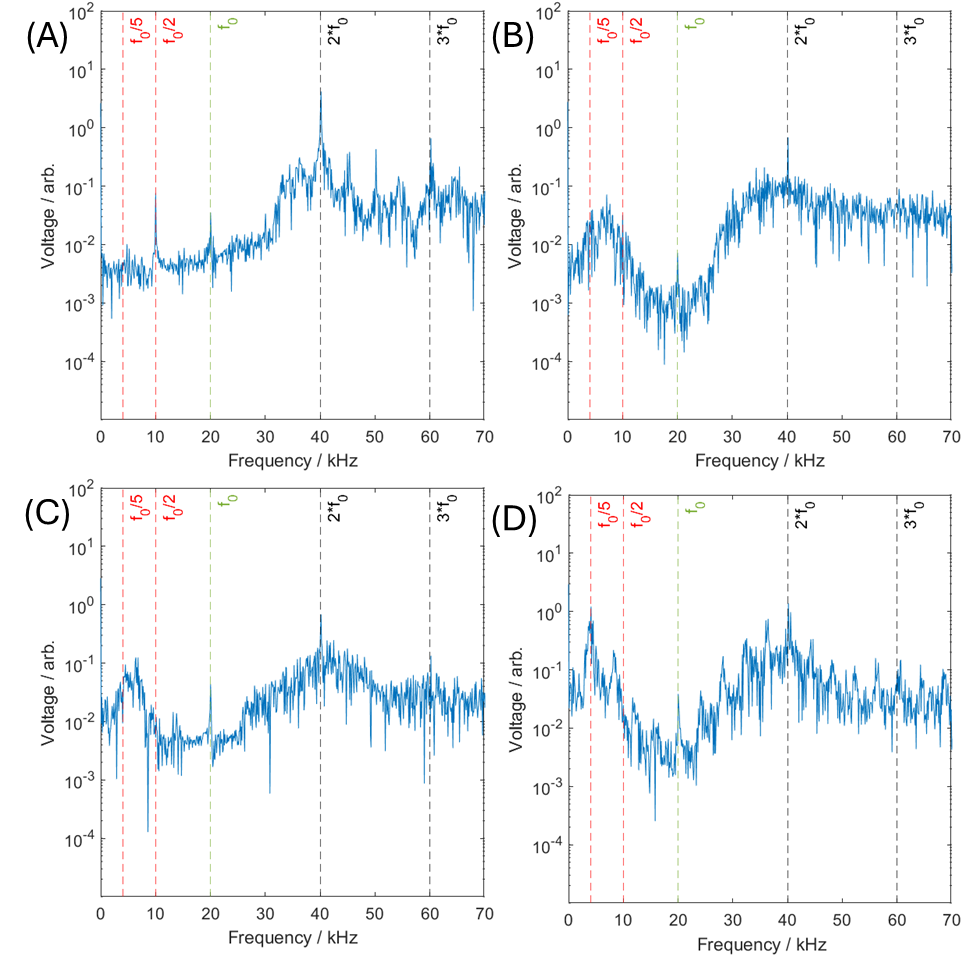
**

**Figure S9**. FFT of acoustic spectra taken with a swPCD of **SiO_2_** with applied ultrasound at tip displacement amplitudes (A) 40 μm (B) 80 μm (C) 120 μm and (D) 152 μm


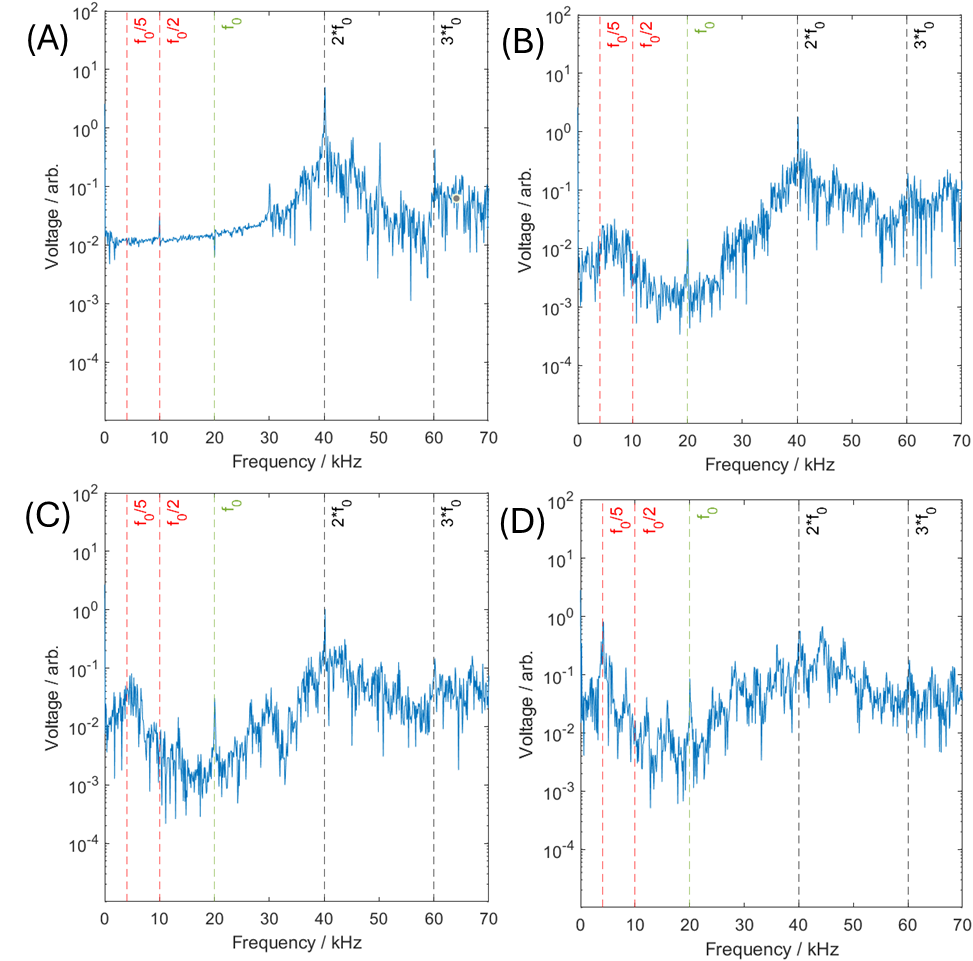


**Figure S10**. FFT of acoustic spectra taken with a swPCD of **CPX**$\boldsymbol{\subset}$**SiO_2_** with applied ultrasound at tip displacement amplitudes (A) 40 μm (B) 80 μm (C) 120 μm and (D) 152 μm


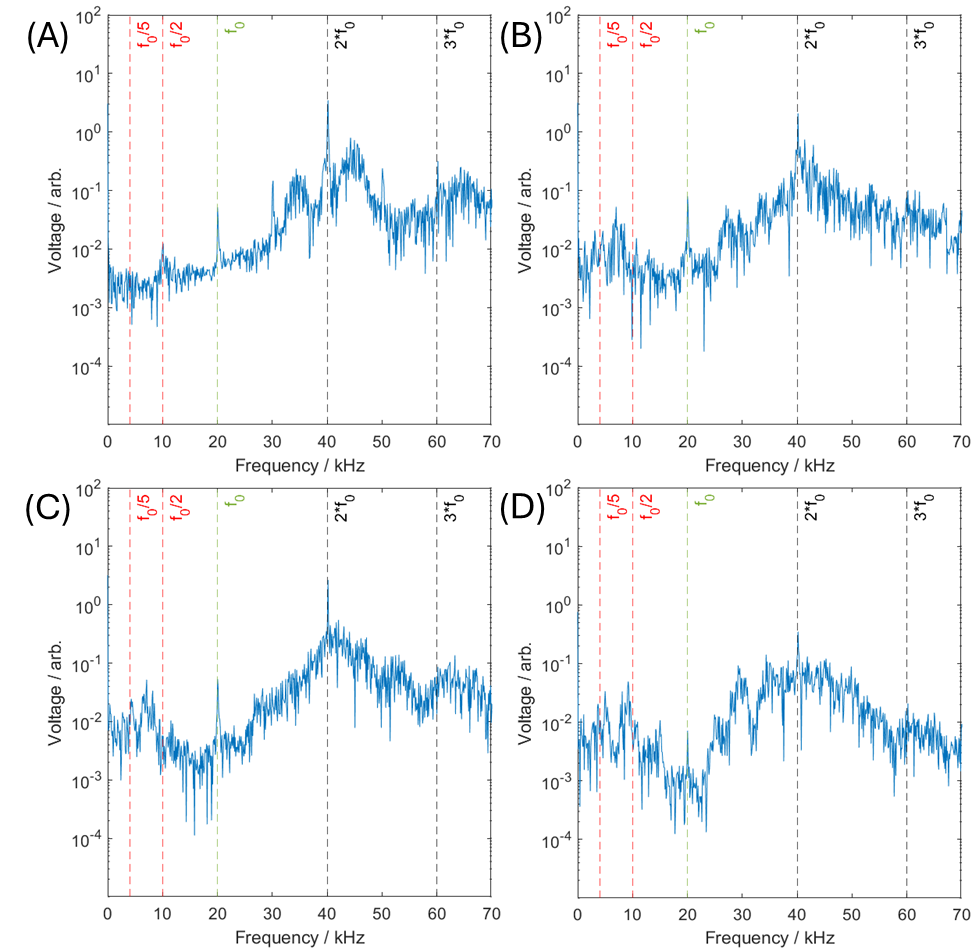
**Figure S11**. FFT of acoustic spectra taken with a swPCD of **m-SiO_2_** with applied ultrasound at tip displacement amplitudes (A) 40 μm (B) 80 μm (C) 120 μm and (D) 152 μm


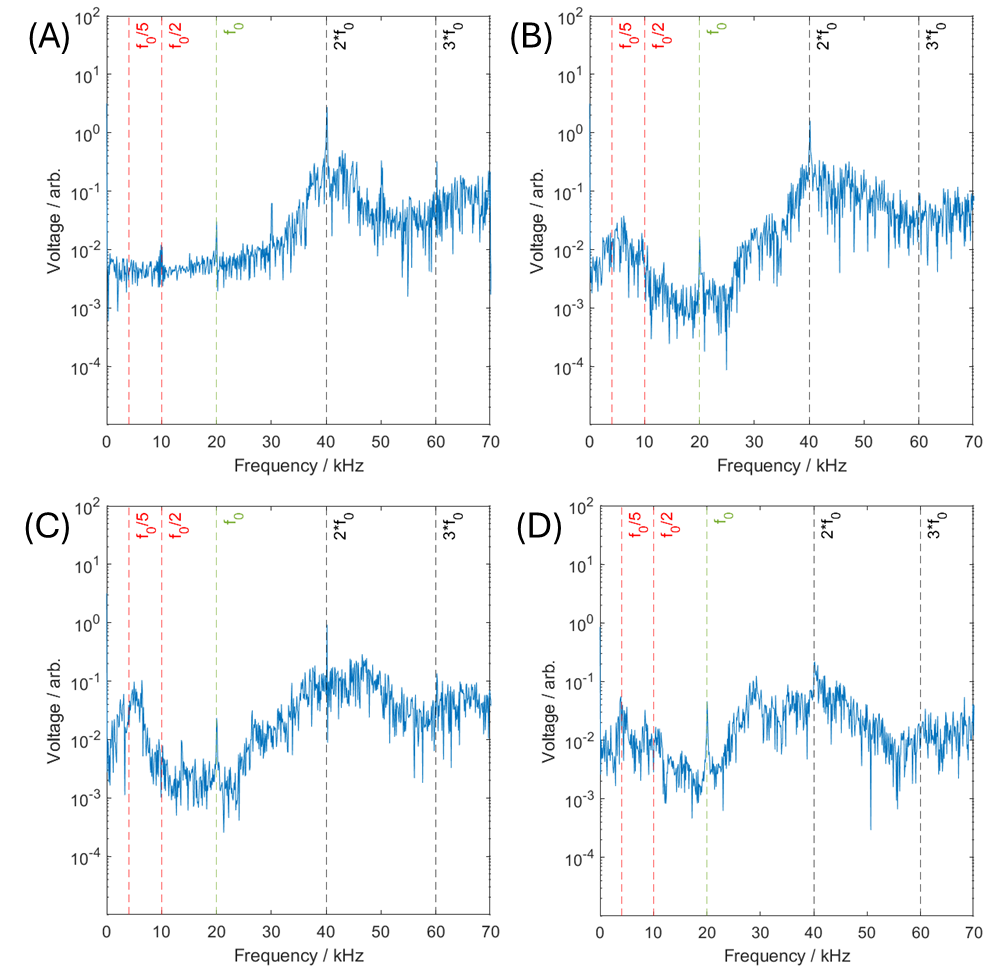


**Figure S12**. FFT of acoustic spectra taken with a swPCD of **CPX@m-SiO_2_** with applied ultrasound at tip displacement amplitudes (A) 40 μm (B) 80 μm (C) 120 μm and (D) 152 μm


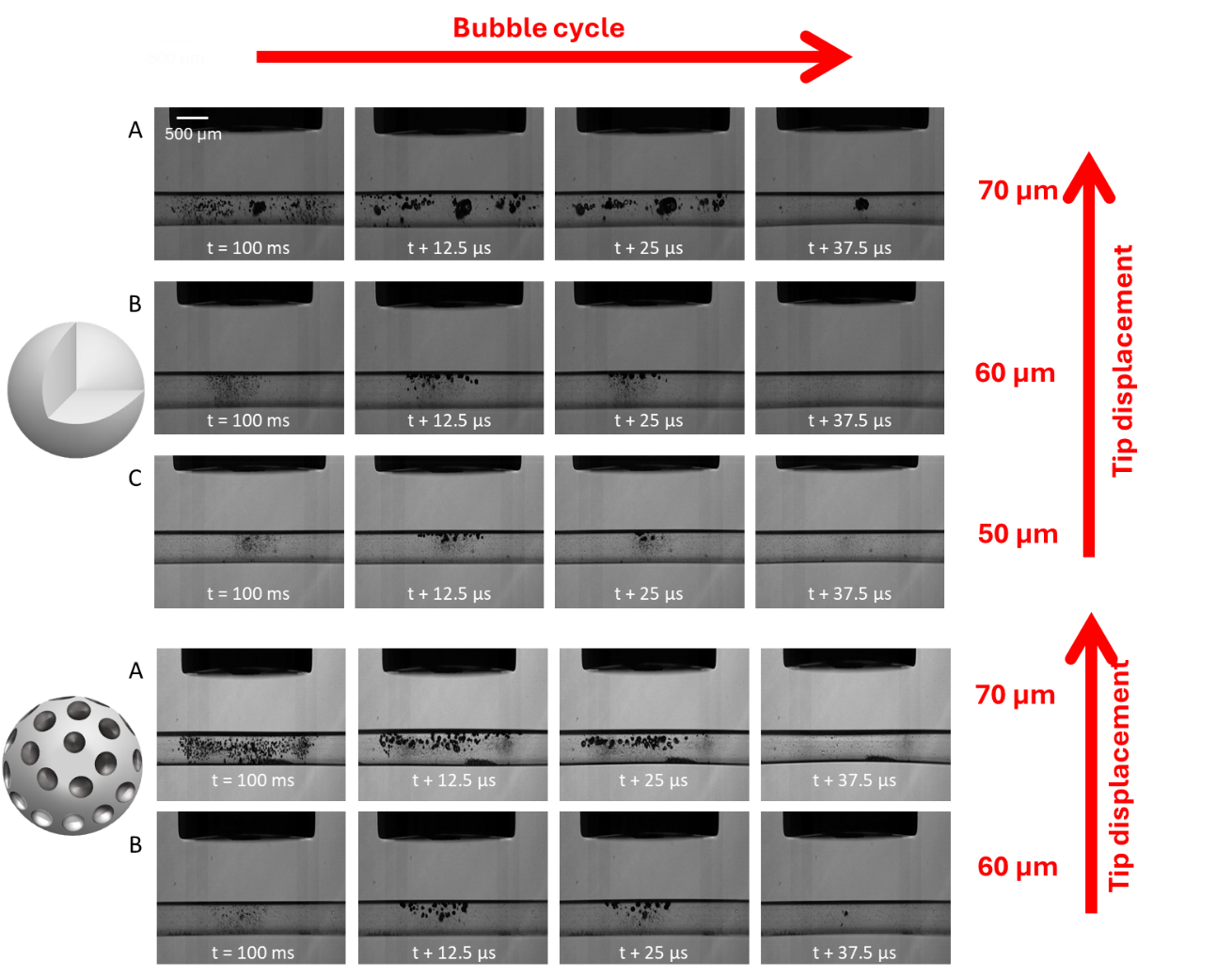


**Figure S13.** Representative Images taken from the capillary high-speed imaging sequences during capillary experiments recorded at 80 kfps such that each row presents one acoustic cycle from a 20 kHz US transducer with **SiO_2_** at A) 70 μm B) 60 μm and C) 50 μm and **m-SiO_2_** at D) 70 μm E) 60 μm
